# Are estimates of intergenerational mobility biased by non-response? Evidence from the Netherlands

**DOI:** 10.1007/s00355-018-1138-0

**Published:** 2018-06-18

**Authors:** Bart H. H. Golsteyn, Stefa Hirsch

**Affiliations:** Department of Economics, Maastricht University, P.O. Box 616, 6200MD Maastricht, The Netherlands

## Abstract

Intergenerational mobility is often studied using survey data. In such settings, selective unit or item non-response may bias estimates. Linking Dutch survey data to administrative income data allows us to examine whether selective responses bias the estimated relationship between parental income and children’s mathematics and language test scores in grades 6 and 9. We find that the estimates of these relationships are biased downward due to parental unit non-response, while they are biased upwards due to item non-response. In the analyses of both unit and item non-response, the point estimates for language and mathematics test scores point in the same direction but only one of the two relationships is significant. These findings suggest that estimates of intergenerational mobility based on survey data need to be interpreted with caution because they may be biased by selective non-response. The direction of such bias is difficult to predict a priori. Bias due to unit and item non-response may work in opposing directions and may differ across outcomes.

## Introduction

The relationship between parental income and children’s schooling outcomes is often estimated using survey data (see, e.g., Blau 1999; Chevalier et al. 2013; Plug and Vijverberg 2005).^1^ A potentially important issue in the literature on intergenerational mobility is that survey response is potentially not random. Most notably, the probability of responding to a survey has been shown to be positively related to income and other indicators of socio-economic status (see the literature review below). An unresolved question is whether non-response biases the estimated intergenerational relationship between parental income and child schooling.

In this study, we examine whether survey-based estimates of the relationship between household income and children’s performance in school are biased because of selective non-response. We focus on two sources of non-response: (1) individual unit non-response by parents unwilling to participate in a survey, and (2) item non-response by parents on questions regarding their income.

We combine parental survey data with administrative data from schools and income register data. Our analysis is based on an ongoing regional education survey conducted in the south of the Netherlands covering more than 95% of primary schools in the target region. Parental survey information is available for approximately 69% of children. We link the data relating to the children to the administrative income data of their parents, which are drawn from Statistics Netherlands. This source provides us with information on parental income for those who respond and do not respond to the survey. Our variable of interest—children’s school performance on standardized mathematics and language tests in grade 6 and 9—is based on the schools’ administrative records.

All our estimations are performed using administrative data only, from schools as well as the income register. We distinguish different types of non-response biases by using observed survey response behavior to artificially restrict the full sample to the data that would be available had the survey suffered from a certain type of non-response.^2^ Observing non-responding households using administrative information enables us to directly assess whether response rates are selective and whether the relationship between parental income and child school performance is biased.

Our main findings are, firstly, that unit non-response at the household level attenuates the intergenerational relationship. Secondly, we find evidence that item non-response on the income question leads to an overestimation of the relationship between parental income and children’s test scores. In the analyses of both unit and item non-response, the point estimates for language and mathematics test scores point in the same direction, but only one of the two relationships is significant.

This study contributes to the literature on intergenerational mobility, specifically the empirical literature that analyzes how children’s schooling outcomes relate to parental income.^3^ Cross-sectional surveys find a consistently significant positive relationship between parental income and schooling outcomes (Blau 1999; Chevalier et al. 2013). Also, when adoption is used as a natural experiment to exclude genetically transferred ability as a factor, the significant relationship with income persists (Plug and Vijverberg 2005). This result remains robust when controlling for characteristics of biological parents and extending the focus to the economic outcomes of the children (Björklund et al. 2006). Hence, parental income appears to be an important determinant of success in school and the labor market.

While findings on the direction of the relationship between parental income and children’s outcomes are unambiguous, their magnitude is often contested. There are several potential empirical problems in studies based on survey data: e.g., non-representative samples, truncated samples, survey non-response, and item-based non-response. Many samples are non-representative by construction, but truncations could also be the result of a choice, such as excluding observations with low or no income. The latter likely leads to overestimations of intergenerational correlations (Couch and Lillard 1998). Unit non-response is inevitable and ubiquitous in survey studies: virtually all self-reported measures of income are subject to item-based non-response.^4^ The question, both with respect to unit and item non-response, is whether the non-response is selective and if so how this may bias the estimated intergenerational relationship.

Earlier research has shown that unit and item non-response are selective in the sense that they are related to income. According to the literature on social-desirability bias, income is a sensitive topic that many survey respondents do not want to speak about. Tourangeau and Yan (2007) show, for instance, that item non-response on income questions is higher than on questions related to sexuality. Bollinger and Hirsch (2013) report that item non-response is higher on income questions than on other questions in the Current Population Survey. Lillard et al. (1986) show that non-response across the earnings distribution is U-shaped, i.e., that respondents in the tails are least likely to report earnings.^5^ These findings are confirmed by, among others, Bollinger et al. (2017) and Bee et al. (2016), who compare survey responses with administrative data.^6^ A valuable point to mention here is that administrative data do not necessarily provide true information on income, as they could, e.g., fail to account for undeclared income.^7^

While there is ample evidence that unit and item non-response are related to income, the issue of whether selective non-response biases estimates of intergenerational mobility has, to our knowledge, not yet received attention in the literature. The contribution of our study is to identify and assess the magnitude of unit and item non-response bias in the context of the intergenerational relationship between parental income and children’s schooling.

The remainder of the study is structured as follows. Section 2 provides a description of the data. Section 3 covers the empirical approach. Section 4 discusses the results. Section 5 highlights potential mechanisms and Sect. 6 concludes.

## Data

We combine different sources of administrative records and survey data. The basis of our dataset is an ongoing regional education monitor for the south of the Netherlands: the OnderwijsMonitor Limburg (OML). The monitor has been conducted as a cooperative project between schools and Maastricht University since 2009. Almost all primary schools in the southern part of the Dutch province of Limburg participate in the project and provide records on their students on a yearly basis.^8^

The data we use are gathered for one cohort of students at two points in time. The students were observed in 2009 in grade 6 (their last year of primary school, completed on average at age 12) and in their third year of secondary school in grade 9 (at about age 15). We give more information about the data below.

At both points in time, a parental survey was conducted and the results were linked to the school records. For grade 6, the parental survey was distributed via the school. In grade 9, the survey was distributed directly to the parents. For the grade 6 data, the overall response rate among the parents amounted to 69%.^9^ Approximately one-third of all non-responding parents had children studying in schools in which none of the parents participated in the survey. This makes it highly likely that those schools either did not send out the survey to the parents or did not forward the completed questionnaires back to the university. In the following, these schools will be referred to as “non-responding schools” so as not to confuse them with schools not participating in the OML. Hence, in our analysis we distinguish non-response at the parental level (22%) and non-response at the institutional or school level (9%). Since non-response at the institutional level is idiosyncratic to our survey design, we discuss the results on this type of non-response in the Appendix. We do provide the main descriptive statistics of this type of non-response in the main text, however.

In the grade 9 survey, the overall parental response rate of 43% is substantially lower than in the survey conducted in grade 6, even though the parental survey was distributed directly to the parents.^10^ The surveys also differ in content. In grade 9, a question regarding household income was included in the parental survey, allowing us to additionally analyze the effect of item non-response on the income questions. Approximately 63% of the responding parents did not fill out the income question.

**Fig. 1:**
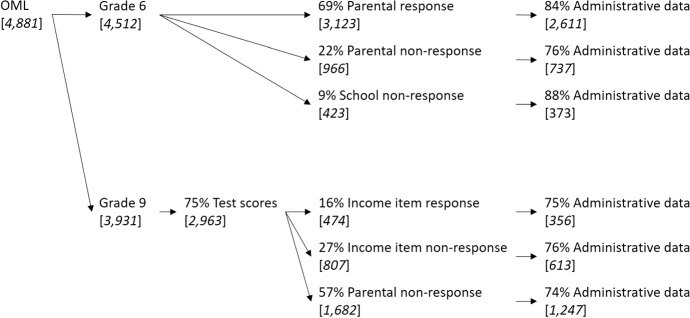
Data collection and response rates in both samples

The process of data collection, as well as the approximate response and non-response rates for both samples, are depicted in Fig. 1. Parental unit non-response can be observed in both samples. Institutional unit non-response can only be assessed in the grade 6 sample and item non-response only in the grade 9 sample.

We merge the data from this cohort to the administrative information from the household register of the municipalities and the income register from Statistics Netherlands performing the match based on surnames and addresses. The match can be completed for 82.5% of all students in the sample assessed in grade 6 and for 74.8% of the sample assessed in grade 9.^11^

### Schooling and income measures

Our measure of school performance in grade 6 is the result of a nation-wide skill assessment test, in the following referred to as the Cito test.^12^ The mean age of the students when they take the test is 12.2 years. The result is the main determinant of teachers’ recommendation for placing students in a certain secondary schooling track, which is often considered binding by secondary schools.^13^ Therefore, the students have strong incentives to score well on this test.

Among grade 9 students, the OML administers its own tests on language and mathematics achievement, based on questions from larger standardized studies.^14^ The results are used solely for research purposes. Hence, in contrast to the Cito test the stakes are low. There are three difficulty levels for the various tracks: one test for the two upper tracks, one for the two medium tracks, and one for the two lowest tracks.^15^ Overlapping questions make it possible to use item response theory (IRT) to transform the raw scores into test scores on a common scale. We use a two-parameter logistic model for binary items, allowing items to vary in their difficulty and discrimination. The resulting latent trait scores are used in the analysis.

The administrative data from Statistics Netherlands on the financial situation of the households comprise measures for total and corrected income. The corrected income is based on total income adjusted for the number of household members. In our analysis, we rely on this corrected measure of household income. This measure comes closer to the income available per child, which for instance can be invested in tutoring.^16^ For each of the income measures, we know to which quintile of the income distribution in the overall population of the Netherlands a household belongs.

Having data on schooling outcomes, as well as an objective measure of household income for an unusually large share of the population, and having a measure for survey participation, are unique features of our dataset. This setting enables us to observe and analyze the non-response bias in the intergenerational relationship of parental income and children’s schooling outcomes.

### Baseline sample, grade 6 (age 12)

The OML covers 95% of schools in the Southern Limburg region. The baseline sample consists of all students attending those schools who took the final assessment test in grade 6 in 2009.^17^ This group consists of 4512 observed students. As mentioned, 69% of their parents (3123) answered the parental questionnaire. For 1389 students no parental information is available. This latter group is split into a group of parents whose child is in a school where none of the parents responded (423) and a group of parents who did not respond, but whose child attended a school in which other parents responded (966). The former group we consider to be missing due to institutional unit non-response, and the latter to be missing due to individual unit non-response. Administrative data on income could be merged for 83.6% of the children with participating parents, and for 79.9% of the children with non-participating ones (76.3% in case of parental unit non-response and 88.2% in case of institutional non-response). Reasons for the imperfect match of administrative data to the OML data include missing or erroneous address data from school records.

Table 1 provides descriptive statistics for all students in the OML and administrative data. Students of non-participating parents and at non-participating schools are slightly older, and among them a greater proportion are first- or second-generation immigrants. Both differences are significant at the 5% level. These descriptive statistics already suggest that distinct selection mechanisms may be at work at the institutional and individual level. With respect to socio-economic status, household income, and student outcomes, there is neither significant negative selection of parents into individual unit non-response, nor of schools into institutional non-response.

**Table 1:** Descriptive statistics for students by survey response status, baseline sample grade 6 (age 12)

Survey participation status										(l) vs. (2)	(l) vs.(3)	(2) vs (3)
Variable	(1)			(2)			(3)					
	Survey participation			School non-response			Parental non-response					
	N	Mean	sd	N	Mean	sd	N	Mean	sd			
Age (in years in grade six)	2611	12.21	.54	373	12.27	.55	737	12.27	.54	**	***	
Gender (fraction male)	2722	.49	.50	429	.48	.50	780	.49	.50			
Low ses (fraction)	2611	.12	.33	373	.10	.31	737	.19	.39		***	***
Migration background (fraction)	2722	.14	.35	428	.20	.40	777	.22	.42	***	***	
Highest parental education (1–5)	2681	3.25	1.09	0	.	.	0	.	.	.	.	.
Corrected HH Income (1–5)	2709	3.05	1.32	426	3.13	1.36	767	2.81	1.35	***	***	***
Cito test score (500–550)	2611	536.90	8.86	373	537.32	9.14	737	534.93	9.06	****	***	***
Cito test: language section (%)	2611	77.19	11.16	373	77.88	11.64	737	74.85	11.70	***	***	***
Cito test: mathematics section (%)	2611	73.50	16.95	373	73.74	16.98	737	70.64	17.00	***	***	***
General cognitive ability (0–43)	2655	32.69	4.68	22	28.91	6.08	556	31.82	5.07	***	***	***
	2722			429			780					

The table reports the number of observations, the mean values and the standard deviations for the listed variables by response status on the parental survey in grade 6 at age 12, conditional on having school administrative information on the students and matched public administration information. The analysis is based on those students for which all variables are available; the most restrictive being the Cito test scores. The different sub-samples (1), (2), and (3) are mutually exclusive. Age is based on the students’ month-exact age at the beginning of grade 6. Gender takes the value of 1 for male students and 0 otherwise. “Low ses” is an indication used and provided by the school administration. Schools receive extra funding if the proportion of students indicated as “low ses” exceeds a certain threshold. It is 1 if one or both of the parents did not complete secondary education (this indication is comparable to the “free lunch” indication in the US). A child is considered to have a migration background if at least one parent was not born in the Netherlands. Parental education level relies on a self-reported measure and is therefore only available if the parental survey was completed. It is coded in terms of the five levels defined in the International Standard Classification of Education (ISCED). Corrected income is reported on the household level in terms of quintiles relative to the entire Dutch population. It is based on the income register at 31 December 2008. The Cito test score reflects the overall score including sections on language, mathematics and study skills. The information provided on the language and mathematics sub-sections are reported in the percentage of questions answered correctly. Dots indicate that the data source of this variable is not available for the group in question. Significance levels are reported as follows: .1*, .05**, and .01***

**Fig. 2:**
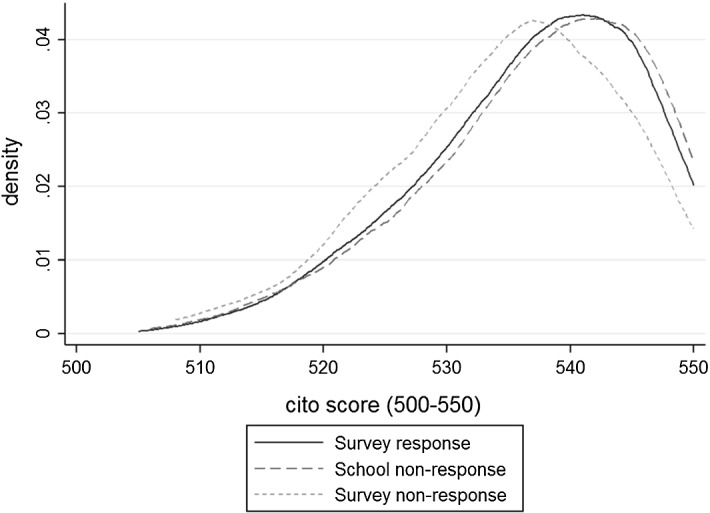
Students’ Cito score distributions by survey response status, baseline sample grade 6 (age 12). This graph is restricted to all students for whom Cito test scores were available and data could be merged with administrative household information. The group with responding parents relates to column (1) from Table 1, the groups with non-responding schools and from non-responding parents correspond to columns (2) and (3), respectively

Distribution plots of Cito scores confirm the differential performance of student groups based on the response to the parental survey. Figure 2 reveals that students whose parents decide to participate in the survey indeed outperform students whose parents decide not to (significant at the 1% level). However, the graph also shows that students attending schools which did not send out the parental surveys perform slightly better on the Cito test than students whose parents responded (significant at the 10% level). Because the Cito test is the main determinant of track placement, these differences potentially also matter for long-run outcomes (College voor Toetsen en Examens 2015).

Table 2 shows parental survey participation rates by quintiles of corrected income. The quintiles are based on the income distribution of the full Dutch population. While parental non-response is highest in the lower income quintiles, school non-response is most prevalent among families in the higher income quintiles. No income records were available for 29 of the 3931 households.

**Table 2:** Parental survey participation by income quintile, baseline sample grade 6 (age 12)

Standardized HH income (quintile)	Survey participation status			Total
	Survey participation	Sccedhool non-resp.	Parental non-resp.	
Lowest	423 15.61	73 17.14	170 22.16	666 17.07
Second	582 21.48	65 15.26	170 22.16	817 20.94
Third	609 22.48	105 24.65	166 21.64	880 22.55
Fourth	632 23.33	98 23.00	154 20.08	884 22.66
Highest	463 17.09	85 19.95	107 13.95	655 16.79
Total	2709 100.00	426 100.00	767 100.00	3902 100.00

The table shows survey participation rates by quintile of corrected income for the three groups distinguished in Table 1. It includes all observations for which data from school administration and Cito test scores are available and can be linked to administrative records of the parents

One caveat in our analysis is that selection may be induced in our sample by an imperfect match with public administrative data. Table 7 provides descriptive statistics of the group for which we do not have administrative data, analogue to Table 1. The table indicates that there is positive selection into the matched sample. Those with available administrative data have higher Cito scores, their socio-economic status is higher, they are less often immigrants, and their parental education level is higher.^18^ Comparing the differences regarding descriptive statistics by participation status, we observe similar patterns in the matched and unmatched sample, especially regarding students’ age and performance: students with non-participating parents and at non-participating schools are slightly older, children at non-responding schools perform best, while children of non-responding parents perform worst.

### Sample, grade 9 (age 15)

The OML also monitors students at grade 9. The cohort observed in grade 6 in 2009 was the first to also be observed in grade 9, the third year of secondary school. In grade 9, a parental survey was conducted and achievement tests in language and mathematics, this time with low stakes for students, were performed. We use this sample to repeat our analysis of parental non-response and explore the influence of item non-response on income questions.

When we restrict ourselves to those participating in the achievement tests, the remaining sample we were able to identify based on names and addresses consists of 2963 observed students. Since the parental surveys are sent out directly, the response rate (43.2%) in this sample solely reflects parental participation and is not influenced by school participation. The survey allows us to examine item non-response on questions regarding household income. Among the survey respondents, 37% answered the income question.

Table 3 provides descriptive statistics for the students in this sample, in analogy to Table 1. Columns (1) and (3) include children of parents who returned the survey, distinguished by whether they provided information on household income (N = 474) or not (N = 807). Column (2) shows the descriptive statistics for all children in grade 9 who participated in the achievement test but whose parents do not return the survey (N = 1682).

**Table 3:** Descriptive statistics for students by survey response status, sample grade 9 (age 15)

Variable	Survey participation status									(l) vs.(2)	(l) vs.(3)	(2) vs. (3)
	(1)			(2)			(3)					
	Survey and item response			Survey non-response			Item non-response					
	N	Mean	sd	N	Mean	sd	N	Mean	sd			
Age (in years in grade six)	474	15.10	.41	1682	15.20	.47	807	15.12	.46	***		***
Gender (fraction male)	474	.46	.50	1682	.49	.50	807	.48	.50			
Low ses (fraction)	455	.04	.50	1595	.17	.38	777	.07	.26	***	**	***
Migration background (fraction)	474	.10	.30	1680	.20	.40	806	.09	.29	***		***
Highest parental education (1–5)	352	3.61	1.03	1093	3.15	1.12	619	3.39	1.00	***	***	***
Corrected HH Income (1–5)	472	3.51	1.24	1669	2.88	1.33	804	3.18	1.27	***	***	***
Cito test score (500–550)	455	539.70	7.82	1595	535.98	8.95	777	537.76	8.63	***	***	***
Language test, grade 9 (stand.)	363	.21	.99	1268	− .08	.96	620	.14	.95	***		***
Mathematics test, grade 9 (stand.)	360	.30	.93	1269	− .13	.97	617	.16	.99	***		***
General cognitive ability (0–43)	399	33.45	4.53	1385	32.33	4.80	690	33.06	4.41	***		***
	474			1682			807					

The table reports the number of observations, the mean values and the standard deviations for the listed variables by response status on the parental survey at age 15, conditional on taking either a mathematics or language test. The different sub-samples (1), (2), and (3) are mutually exclusive. Age refers to the students’ month-exact age at the beginning of grade 9. The variables gender, low ses, migration background, parental education, income and Cito test were assessed in grade 6 and are the same as in Table 1. Gender takes the value of 1 for male students and 0 otherwise. “Low ses” is an indication used and provided by the school administration. Schools receive extra funding in case the proportion of students indicated as “low ses” exceeds a certain threshold. It is 1 if one or both of the parents did not complete secondary education (this indication is comparable to the “free lunch” indication in the US). A child is considered to have a migration background if at least one parent was not born in the Netherlands. Parental education level relies on a self-reported measure and is therefore only available if the parental survey was completed. It is coded in terms of the five levels defined in the International Standard Classification of Education (ISCED). Corrected income is reported on the household level in terms of quintiles relative to the entire Dutch population. It is based on the income register at 31 December 2008. The Cito test score reflects the overall score including sections on language, mathematics and study skills. The variables language test and mathematics test refer to scores on tests conducted specifically for the OML. The reported scores are standardized to a mean of zero and a standard deviation of one. Significance levels are reported as follows: .1*, .05**, and .01***

Children of parents who do not respond to the survey are slightly older and substantially more likely to be first or second-generation immigrants. With respect to parental socio-economic status and income, as well as all measures of students’ school performance, the group of parents who do not respond has unfavorable characteristics. Within the group of respondents, parents who answer the income question have on average more favorable characteristics. Their children also perform better on all tests considered.

Distribution plots for the language and mathematics tests (see Figs. 5, 6) show that children of responding parents clearly outperform children of non-responding parents (significant at 1%). The distributions are similar for children with responding parents who do or do not answer the income question.

Table 4 reveals that the survey non-response rate is higher at lower income quintiles. In turn, the item non-response rate is lowest among parents in the lowest income group. No income measure is available in the income registry for 18 of the 2963 households.

**Table 4:** Parental survey participation by income quintile, sample in grade 9 (age 15)

Standardized HH income (quintile)	Survey participation status			
	Survey and item participation	Survey non-resp.	Item non-resp.	Total
Lowest	33 6.99	324 19.41	92 11.44	449 15.25
Second	75 15.89	371 22.23	174 21.64	620 21.05
Third	107 22.67	394 23.61	175 21.77	676 22.95
Fourth	130 27.54	337 20.19	222 27.61	689 23.40
Highest	127 26.91	243 14.56	141 17.54	511 17.35
Total	472 100.00	1669 100.00	804 100.00	2945 100.00

The table shows survey and item response rates by quintile of corrected income for the three groups distinguished in Table 3. It includes observations for which data from school administration and test scores are available and which can be linked to administrative records of the parents

## Empirical approach

Our empirical approach is to analyze regressions with children’s schooling outcomes as dependent variables and parental income as the independent variable. This relationship between parental income and children’s educational outcomes is a correlation and we do not attempt to estimate the causal relationship. There are many factors which may potentially explain the relationship, e.g., parental IQ may be related both to parental income and children’s educational outcomes. In our analysis, we examine whether the correlation between parental income and children’s educational outcomes is biased due to selective survey participation.

Based on administrative data only (for test scores, household income, and control variables), we explore the relationship between parental income and students’ academic performance as a measure of intergenerational mobility. We use parental surveys solely to construct hypothetical sub-samples, which suffer from different types of non-response. We run the same regressions based on responding and non-responding sub-groups. Comparing the coefficients for parental income between these regressions provides insight into whether a certain type of non-response biases the estimated relationship between income and schooling outcomes.

In the literature, the most commonly used measure of intergenerational mobility is the elasticity in a regression of the log of child income on the log of parental income. Authors typically do not add controls to this regression (see, e.g., Jäntti and Jenkins 2015; Corak 2013; Nybom and Stuhler 2015). They instead estimate the intergenerational mobility separately for boys and girls and measure lifetime income at specific ages of the parent and child. Our approach differs in some regards from the standard approach. First, we look at children’s educational outcomes rather than their income. Second, we control for various characteristics of the child (age, gender, indicators of low socio-economic status, and whether the child is a first or second-generation immigrant) instead of running the estimates separately for various characteristics.^19^ In the Appendix, we also show the main tables of the paper without controls (Tables 13, 14). The results are qualitatively robust to this exclusion. In a quantitative sense, all coefficients become somewhat larger in the analyses without controls than in the analyses with controls.

## Results

In this section, we analyze unit non-response bias and income item non-response. Furthermore, we report robustness checks.

### Parental unit non-response in grade 6

In the sample assessed in grade 6 (age 12), we analyze individual unit non-response by parents. As mentioned, the results on institutional unit non-response by schools are displayed in the main tables but discussed in the Appendix.

**Fig. 3:**
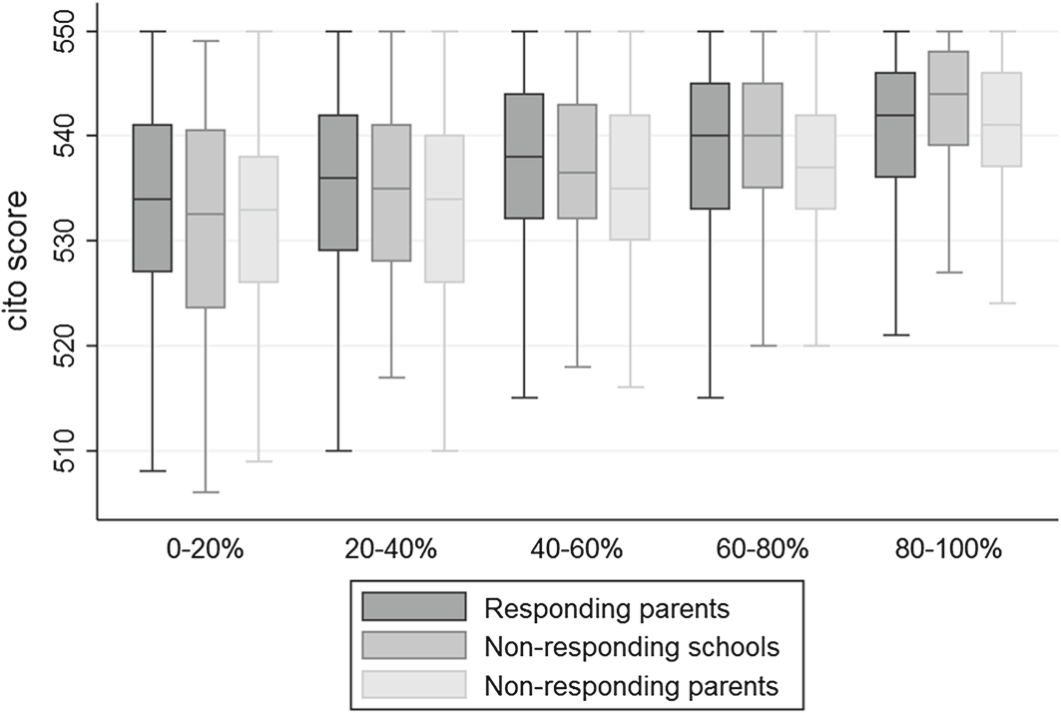
Box plots of students’ Cito scores by survey response and income, baseline sample grade 6 (age 12). Box plots for Cito test results, displayed by survey response status and income quintile. The boxes are drawn around the median, indicated by the line in the box, and show the interquartile range from the 25th percentile to the 75th percentile. The whiskers show the span of the data points. Their maximum length is 1.5 times the interquartile range. Data points outside this span are usually displayed separately. In accordance with the policy on non-disclosure of individual data by Statistics Netherlands, the figure does not include those outside values (here: 24 observations). The graph is based on 2602 students whose parents participated in the survey, 726 students whose parents did not respond, and 371 students whose schools did not respond

The box plots in Fig. 3 provide a first insight into the test score distributions by income group. They allow a comparison of the median, the 25th and 75th percentile, as well as the span of Cito scores, between the different subgroups by response status. Differences between the groups can be observed for all income quintiles. Across all income quintiles, the median student whose parents do not respond performs worse than the median student whose parents respond. The same holds at the 75th percentile and, in the three lowest income quintiles, also for the 25th percentile of the distributions. A Kolmogorov–Smirnov test on the underlying distributions shows that this difference is significant for the third and fourth income quintile. Figure 3 confirms the finding shown in Table 1 that children of non-responding parents perform significantly worse than those of responding parents. However, these differences could be explained by differences with respect to background characteristics or income levels. In the following, we therefore explore whether the gap in schooling outcomes between children of responding and non-responding parents remains after controlling for demographics, and income. As a basic set of controls, we use students’ gender and age as well as an indicator for low socio-economic status.^20^

Table 5 shows the results of the main regression specifications, displaying only the income coefficients. We run the same regression of students’ performance on the parental income quintile and control (1) for the full sample and (2) for a number of artificially restricted samples. In a first step, the full sample is split into students attending a non-responding school (NRS) or a responding school (RS). In a second step, the sub-sample attending responding schools is split into parental response status. This allows us to compare children of non-responding parents (NRP) to those of responding parents (RP).

**Table 5:** Relationship between students’ test scores and parental income, different samples (grade 6)

	N	Coefficients for income quintile		
		(1) Cito test	(2) Language	(3) Mathematics
Full sample	3699	.213*** (.000)	.205*** (.000)	.171*** (.000)
Non-responding schools (NRS)	371	.284*** (.000)	.276*** (.000)	.228*** (.001)
Responding schools (RS)	3328	.204*** (.000)	.196*** (.000)	.165*** (.000)
Non-responding parents (NRP)	726	.217*** (.000)	.209*** (.000)	.168*** (.000)
Responding parents (RP)	2602	.196*** (.000)	.187*** (.000)	.160*** (.000)

Wald test on income coefficients	Hypothesis	p values		
School non-response	NRS = RS	.116	.035**	.173
Parental non-response	NRP = RP	.502	.474	.758

The table reports OLS regressions of children’s Cito test scores on parental income. Each cell represents a separate regression. The dependent variables are total Cito scores as well as percentile scores on the sub-sections language and mathematics, standardized to a mean of zero and a standard deviation of one. The coefficients shown in each cell are those of the independent variable income, which is measured in quintiles, based on the Dutch population. Income refers to corrected income, adjusted for the number of household members. Each regression controls for a number of variables: age, gender, and indicators of low socio-economic status as well as of first- or second-generation immigrant status. Standard errors are reported in parenthesis. They are clustered at the school level (there are 174 schools). Significance levels are reported as follows: .1*, .05**, and .01***

In this setup, comparing the NRP and RP samples shows the bias induced by individual unit non-response.^21^ We repeat this analysis for three different schooling outcomes: (1) the total Cito score, (2) the fraction of correct tasks in sub-sections for language, and (3) the fraction of correct tasks in sub-sections for mathematics. All of these measures are standardized to a mean of zero and a standard deviation of one. Thus, the resulting coefficients can be interpreted as a change in standard deviations, associated with an increase of one quintile in income. P-values for the differences in the estimated income coefficients, between non-responding and responding parents, are provided in the second panel of Table 5.

All coefficients of the relationship between parental income quintile and students’ schooling outcomes are significant at the 1% level.^22^ Regarding all three performance measures, the coefficients for the sample with children of responding parents exceeds that for the sample with children of non-responding parents in magnitude. However, none of these differences are significant. A similar empirical approach, based on the full sample and the use of interaction terms between different types of non-response and the income quintile, confirms these results (see Table 8). The only significant differences between coefficients of intergenerational mobility in this sample are found when analyzing institutional non-response (see Appendix).

### Parental unit non-response and item non-response in grade 9

We apply the same empirical strategy to the sample assessed in grade 9 (average age 15). In this wave, the parental survey was sent out directly to parents, providing a direct measure of parental survey response. This parental survey included a question regarding household income, allowing us to further assess a potential bias due to specific item non-response.

In the following, we distinguish three groups: (1) children of parents who responded to the survey and provided a measure of household income, (2) children of survey responders who did not provide an income measure, and (3) children of survey non-responders.

**Fig. 4:**
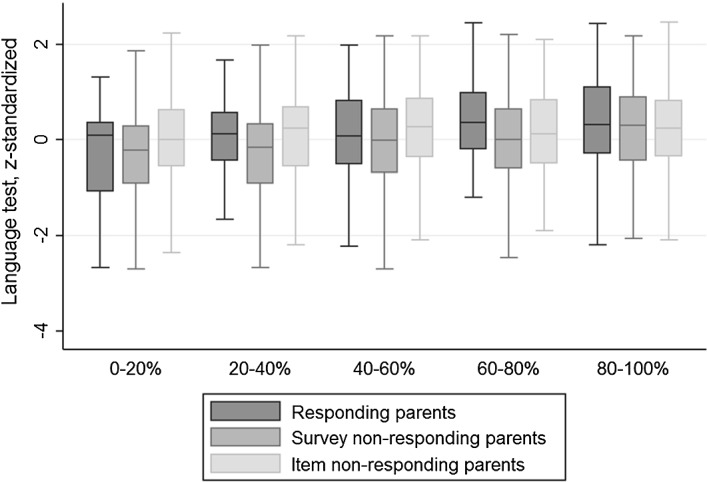
Box plots for students’ language test by survey response and income, sample grade 9 (age 15). Box plots for language test results in grade 9, displayed by survey response status and income quintile. The boxes are drawn around the median, indicated by the line in the box, and show the interquartile range, from the 25th percentile to the 75th percentile. The whiskers show the span of the data points. Their maximum length is 1.5 times the interquartile range. Data points outside this span are usually displayed separately. In accordance with the policy on non-disclosure of individual data by Statistics Netherlands, the figure does not include those outside values (here: 34 observations). The graph is based on 356 students whose parents participated in the survey, including the income question, 613 students whose parents responded to the survey without reporting household income and 1247 students whose parents did not return the survey

**Fig. 5:**
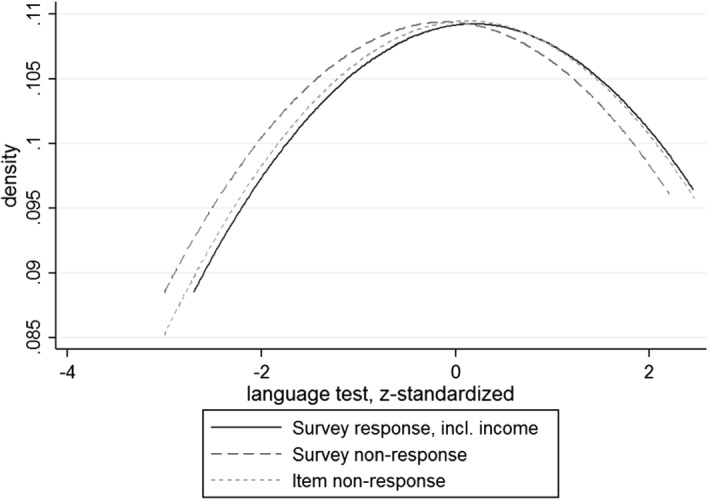
Students’ language test distributions by survey response status, sample grade 9 (age 15). This graph shows the distribution of students’ language achievement test, standardized to a mean of zero and a standard deviation of one. It is based on all grade 9 students for whom data could be merged with administrative household information and who took the language achievement test. The group with responding parents who answered the income question relates to column (1) from Table 3, the group with non-responding parents correspond to column (2), and responding parents who do not answer the income question, the item non-response group, relate to column (3)

**Fig. 6:**
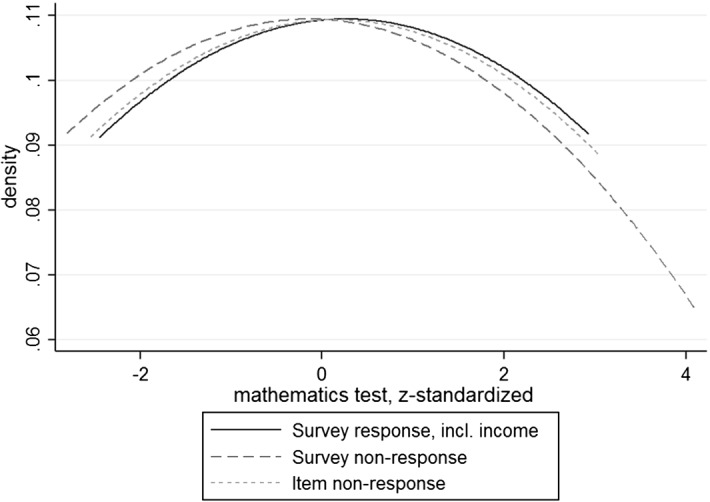
Students’ mathematics test distributions by survey response status, sample grade 9 (age 15). This graph shows the distribution of students’ mathematics achievement test, standardized to a mean of zero and a standard deviation of one. It is based on all grade 9 students for whom data could be merged with administrative household information and who took the language achievement test. The group with responding parents who answered the income question relates to column (1) from Table 3, the group with responding parents who do not answer the income question, non-responding parents correspond to column (2), and the item non-response group, relates to column (2)

**Fig. 7:**
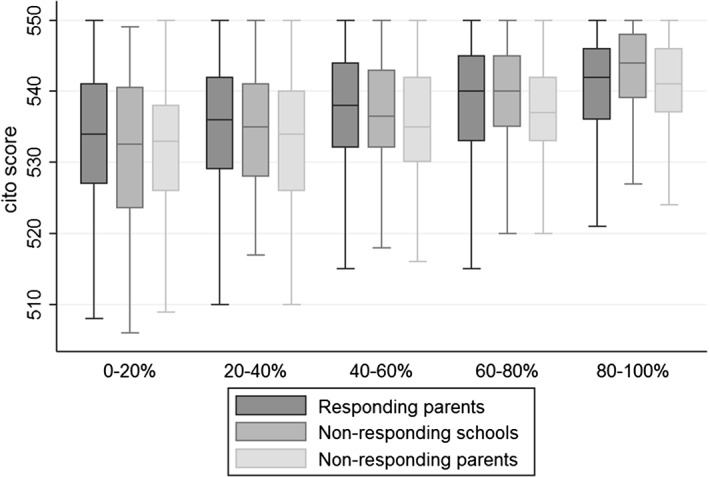
Box plots for students’ mathematics test by survey response and income, sample grade 9 (age 15). Box plots for mathematics test results in grade 9, displayed by survey response status and income quintile. The boxes are drawn around the median, indicated by the line in the box, and show the interquartile range, from the 25th percentile to the 75th percentile. The whiskers show the span of the data points. Their maximum length is 1.5 times the interquartile range. Data points outside this span are usually displayed separately. In accordance with the policy on non-disclosure of individual data by Statistics Netherlands, the figure does not include those outside values (here: 20 observations). The graph is based on 425 students whose parents participated in the survey, including the income question, 717 students whose parents responded to the survey without reporting household income and 1527 students whose parents did not return the survey

The box plots presented in Fig. 4 show how achievement in the language test is distributed within the different income quintiles, separately by parental response status. In general, the graph reveals that the differences by participation status based on this test seem to be smaller than the differences based on the Cito test in grade 6 (compare to Fig. 3). A tendency towards positive selection into survey response is observed. However, among children of survey responders, particularly children of low-income parents who do not respond to the income question, outperform the children of those who do respond, at most points in the distribution. The analog box plots for achievement in the mathematics test in grade 9 are provided in the Appendix, in Fig. 7. Figs. 5, 6 additionally show the corresponding distribution plots by response status.

Applying the same approach used in the baseline sample, we examine whether unit and item non-response bias the estimated relationship between parental income and children’s schooling. Table 6 displays the income coefficients of the main regression specification for the full sample as well as artificially restricted sub-samples.

**Table 6:** Relationship between students’ test scores and parental income, different samples (grade 9)

		Coefficients for income quintile			
		(1)		(2)	
		Language	N	Mathematics	N
Full sample		.122*** (.000)	2216	.152*** (.000)	2198
Survey non-responding parents (SNR)		.128*** (.000)	1247	.167*** (.000)	1241
Survey responding parents (SR)		.087*** (.001)	969	.093*** (.000)	957
Item non-responding parents (INR)		.042 (.191)	613	.080** (.011)	605
Survey, and item responding parents (IR)		.164*** (.000)	356	.102*** (.005)	352

Wald test on income coefficients	Hypothesis	p values	
Parental survey non-response	SNR = SR	.225	.020**
Parental item non-response	INR = IR	.017**	.643

The table reports OLS regressions of children’s language and mathematics scores on parental income. Each cell represents a separate regression. The dependent variables are the fraction of correctly answered questions on the tests, standardized to a mean of zero and a standard deviation of one. The coefficients shown in each cell are those of the independent variable income, which is measured in quintiles, based on the Dutch population. Income refers to corrected income. Each regression controls for a number of variables: age, gender, and indicators of low socio-economic status as well as first- or second-generation immigrant status. Standard errors are reported in parenthesis. They are clustered at the school and track level (there are 579 school-track combinations). Significance levels are reported as follows: .1*, .05**, and .01***

First, children are split according to whether their parents did (SR) or did not respond to the survey (SNR). Then, children of survey respondents are distinguished by whether the parents did (IR) or did not fill in the included income question (INR). Comparing the coefficients of the SNR and SR samples provides a measure for unit non-response bias. A comparison between the INR and IR samples provides insight into whether item non-response induces a bias. This analysis is conducted for the language (1) as well as the mathematics achievement test (2), administered in grade 9 as part of the OML.^23^ Both measures are standardized to a mean of zero and a standard deviation of one.

As can be seen in Table 6, the analysis based on the language test confirms the results from the baseline sample for language scores: no significant evidence is found that unit non-response is a source of bias in the estimated relationship between parental income and students’ language outcomes. For mathematics, we do find significant differences. The estimate reveals that the relationship is attenuated due to parental non-response. Regarding item non-response on income questions, we find that the estimated relationship is biased upwards for language test scores. However, the difference is not significant for mathematics test scores.^24^

Surprisingly, the estimated coefficient for parental income in the sample of item non-responding parents is very small and insignificant. This observation may be explained by the contrast between the two tests. The stakes of the Cito test, used in the baseline sample, are high as they partly determine secondary school track placement. By contrast, the tests in grade 9 have no relevance for the students in terms of grades or placement.

The availability of a self-reported and an administrative measure of parental income also allows us to assess reporting bias in an intergenerational mobility setting: using survey-reported income to measure intergenerational mobility (IGM), and then comparing the (potentially) “biased” survey estimates with the “true” estimates using administrative measures of income. The results indicate that the relationship between parental income and children’s educational outcomes is biased when using self-reported measures of income. In our dataset, the relationship between self-reported income and educational outcomes is not significant. Relative to the relationship between administrative measures of income and educational outcomes, we can conclude that the results using self-reported income are attenuated both for language (*p* value .030) and for mathematics (p value .051).

### Robustness checks

All of the above regression results rely on a corrected measure of household income, based on the total household income adjusted for number of household members. As a robustness check, we conduct all analyses also with the measure of total income. The results remain qualitatively similar but are somewhat weaker in magnitude and significance. Since adjustment by number of household members is highly relevant for the actual standard of living, this is not surprising. In support of this, for the sub-sample of parents with a known education level, we also find that the quintile of a household’s corrected income correlates more strongly with highest education level than with total income.

The error terms of the test scores are unlikely to be independent across students in the same environment. Attending the same school and having the same teacher could systematically influence student achievement. Therefore, we account for common factors of influence in the results shown above. To ensure that our results are not driven by clustering, we repeat all analyses with non-clustered standard errors. We do not find any major difference to the results discussed above.

Significant differences in the fraction of immigrants in groups by response status raise the question whether these could drive the reported results. In the Appendix, we show estimates excluding immigrants (Tables 15, 16). The coefficients are somewhat larger compared to the results based on the full sample including immigrants. However, in a qualitative sense, the results on the differences between the sub-groups by response status remain unchanged. Therefore, immigrants do not drive the conclusions we draw.

## Potential mechanisms

Our results indicate that individual unit and item non-response by parents may bias estimates in opposite directions. In the following, we discuss potential mechanisms behind the underlying selection into unit and item non-response in our samples.

### Selection into individual unit non-response

The magnitude of the intergeneration mobility coefficient in the group of responding parents is, across both age groups and for all outcome measures, consistently lower than in the group of non-responding parents. Thus, individual unit non-response could potentially lead to an overestimation of intergenerational mobility.

To get a better insight into the sorting of parents in terms of survey participation, we regress parental survey response on individual student characteristics. The results are presented in the Appendix in Table 11. In both surveys, grade 6 and 9, being a first or second-generation immigrant is associated with a lower probability of parental survey participation. Also, children’s academic performance is related to a higher probability of parental response. In primary school, Cito scores significantly predict parental response. In secondary school, the more recent performance measures are more important. Attending a secondary school track other than the lowest one is associated with a lower response rate among parents. However, this is conditional on all the other variables, and the coefficient on having low-educated parents (“low ses”) is negative and exceeds any of the coefficients on track. Again in the secondary school sample, parents in households not belonging to the lowest or the middle income quintile are more likely to respond to the survey. Effects regarding school location are no longer significant once a variable on the use of dialect is added. In both samples, speaking the local dialect well is associated with a higher probability of parental survey response.

All in all, in both surveys the parents of well-performing students who are not immigrants and who speak the local dialect well are more likely to answer. In the secondary school sample, socio-economic background and track placement play an important role as well.

### Selection into income item non-response

Among parents who return the survey but do not answer questions on household income, the relationship between parental income and children’s language performance is weaker than among parents who do respond to the question. The selection into item non-response could shed some light on the potential mechanisms behind this bias.

While there is a positive selection into unit response with respect to parental education, parental income, and students’ test scores, this is different for item non-response. As can be concluded from the boxplot in Fig. 4 (as well as Fig. 7), children of low income item non-responders, in particular, outperform those of item responders.

An explanation that fits in with this pattern is that parents with low income but otherwise favorable characteristics^25^ may feel that they do not live up to expectations and are therefore reluctant to report their income. This is consistent with non-responders having atypical characteristics for the area they live in, as has been shown for the US census (Bee et al. 2016). Such a systematic item non-response pattern is particularly harmful for estimates of intergenerational mobility. Since the non-responders are atypical for the area they live in, imputation of missing data based on postal code area, as applied by Sabelhaus et al. (2015), is risky.

An additional difference can be observed in the mode of responding to the survey. The majority of item responders (88.9%) filled out the online survey to which a link was provided in the initial invitation letter, while only 22.5% of item non-responders did so. Most of them (77.5%) instead filled out the paper survey which was only provided in the reminder letter. This points towards a potential link between the mode of the survey, the willingness to respond to certain items, and potentially further characteristics.

A regression of parental item response on individual student and parent characteristics can be found in the Appendix in Table 12. Student performance measures only positively significantly predict parental item response if track and school location are not included. Parental item response is dominantly associated with belonging to the highest income quintile^26^ and answering the survey upon the first request online.

## Conclusion

This paper assesses the potential bias in estimates of intergenerational mobility, specified as the relationship between parental income and students’ school performance, combining administrative and survey data. We identify different sources of non-response—parental unit and item non-response—and show that they have to be considered separately because their effects on the estimates of intergenerational mobility are distinct.

Our main results indicate, firstly, that unit non-response at the household level attenuates the intergenerational relationship. Secondly, we find evidence that item non-response on the income question leads to an overestimation of the relationship between parental income and children’s test scores. In the analyses of both unit and item non-response, the point estimates for language and mathematics test scores point in the same direction but only one of the two relationships is significant.

These results are important both for the literature on intergenerational mobility but also for that in other contexts. Non-response is ubiquitous and substantial in all estimations based on survey data, e.g., among the articles we cite in our paper, non-response rates in other published studies on intergenerational mobility are found of up to 42%. Our results suggest that estimates of intergenerational mobility based on survey data need to be interpreted with caution because they may be biased by selective non-response. The direction of such bias is difficult to predict a priori. Bias due to unit and item non-response may work in opposing directions and may differ across outcomes.

Matching survey and administrative data enables us to directly assess the effects of non-response. A remaining downside of our data set is the imperfect match of survey and performance data to administrative data. Even though, at above 80% in the baseline sample, this match is relatively high, it is not perfect. The remaining selection may drive our results to some extent.

To our knowledge, this is the first study on bias induced by selective unit and item non-response in the important relationship between parental income and children’s school performance, as an early measure of intergenerational mobility. Obviously, the results could be driven by the specific setting of our study and as mentioned above, our results may partly be driven by incomplete matching to administrative data. Therefore, this study needs to be replicated in different contexts. This is feasible in countries where large administrative datasets are already available, for example in Scandinavia. The same approach can of course be used to assess the bias induced by non-response in any relationship of interest estimated based on survey data.

## Appendix 1

### Onderwijsmonitor Limburg

The *OnderwijsMonitor Limburg* (OML) is a collaboration between Maastricht University, educational institutions (primary, secondary and tertiary) and government bodies in the Province of Limburg (in the south of the Netherlands).

The OML aims to gain insights into the educational development of pupils/students in order to foster the further improvement of education and the transition from education to the labor market in the Province of Limburg, and to acquire knowledge about the dynamics of educational processes in general.

More than 95% of all primary schools in the south of Limburg participate in this education monitor. The project is approved by the local school boards of Limburg. Test scores are processed by the MEMIC Maastricht (center for data and information management) in anonymized form.

The key elements of the OML are:

- structural accumulation of relevant data throughout the educational career of pupils/students in Limburg (longitudinal)
- effective dialogue and collaboration between the OML partners
- high-quality research (both academic and applied) and dissemination of insights into the domains of academia, policy and educational practice.

The OML collects data regarding:

- cognitive pupil/student outcomes (school career and test results)
- non-cognitive pupil/student characteristics (primarily socio-emotional and socio-economic)
- parent and household characteristics
- teacher characteristics
- school characteristics.

The starting point of the OML is information which is already collected within the schools. Additional testing and surveying is done where needed (in close collaboration with the schools).

Earlier academic publications using data from the OML are:

- Borghans, L., Golsteyn, B., Zölitz, U. (2015). School quality and the development of cognitive skills between age four and six. *PLOS ONE* 10(7), e0129700.
- Feron, E., Schils, T., ter Weel, B. (2016), Does the teacher beat the test? The additional value of the teacher’s assessment in predicting student ability. *De Economist* 164(4), 391–418.
- Golsteyn, B., Schils, T. (2014). Gender gaps in primary school achievement: A decomposition into endowments and returns to IQ and non-cognitive factors. *Economics of Education Review* 41, 176–187.
- Künn-Nelen, A., de Grip, A., Fouarge, D. (2015). The relation between maternal work hours and the cognitive development of young school-aged children. *De Economist* 163(2), 203–232.

## Appendix 2

### Institutional selective non-response

In this appendix, we discuss the results with respect to selective institutional non-response.

Figure 3 shows that students attending non-responding schools mostly outperform students with responding parents at the lower end of the test score distribution. However, in the lowest income quintile, the variance is larger for students in non-responding schools. In the highest income quintile, students at non-responding schools outperform both other groups across the full distribution.

Table 5 reveals that the largest coefficients, i.e., the lowest intergenerational mobility, can be observed for the sample of non-responding schools. A quantitative comparison of the coefficients across the samples shows that only the difference induced by institutional unit non-response is significant. From separate regressions for performance in language and mathematics, it becomes apparent that the bias is more strongly driven by differences in the language sub-section of the Cito test. Table 8 confirms these findings. The interaction term between income quintile and school non-response for total Cito scores or the language sub-section is significant at the 5 and in some cases the 1% level. Again, this is different for mathematics scores. The magnitude of this interaction effect amounts to more than half of the income quintile coefficient. When applying this to the difference between the lowest and the highest income quintile, the gap in the estimates for children of responding parents and non-responding schools amounts to about a third of the standard deviation in Cito test scores. Using probability weights to account for differential participation based on the parental income group cannot correct this bias.

In sum, regarding institutional unit non-response by schools, we find evidence for a significant overestimation of intergenerational mobility.

What are the potential underlying mechanisms? The fourteen schools which did not send out the parental surveys show a stronger relationship between parental income and children’s schooling outcomes than responding schools. This could either be explained by differences in characteristics, by anticipation of parental response behavior, or because the schools may use non-response as a strategy to avoid unfavorable results. These explanations are discussed in the following.

All of the non-responding schools except for one are Roman Catholic, as are the majority of primary schools in the sample. Table 1 shows that significantly fewer students from a low socio-economic background attend non-responding schools, while more first- or second-generation immigrants attend such schools. This combination of characteristics would be consistent with a large fraction of children of academically trained expats in the student population.^27^ Furthermore, while the students at non-responding schools on average score higher on the Cito test, they also have a higher standard deviation. The same holds for parental income. With the favorable selection of student population and high average performance, non-responding schools are unlikely to attract the attention of the Inspectorate of Education. However, they seem to be of low quality in terms of addressing intergenerational mobility.

Should the schools be aware of this, a potential explanation for their non-response could be that they do not want to disclose this weakness. Similarly, the extent of participation in the research project could be positively related to how much schools care about equality of opportunity. This comparison of responding and non-responding schools is purely descriptive and unconditional. A regression of school participation on aggregated characteristics (see Appendix, Table 10) does not show any significant relationship with the above-mentioned characteristics. However, schools in two of the regions are significantly more likely to participate.

School non-response could also be driven by anticipated parental non-response. That is, schools may not respond because they assume that parents would not be willing to participate in the surveys. Since the survey in grade 9 is sent out directly to the same parent population, we can check whether there is a basis for this assumption. The differences in parental response behavior that we find are very small. Parents whose children attended a non-responding primary school seem to be even more willing to participate in surveys and to provide complete information. So if schools do not respond because they thought parents in the school would not want to, their assumption is incorrect.

The following patterns with respect to school response can be observed: especially schools with high average test scores, but also high intergenerational dependence, do not send out surveys to parents. These schools have on average a lower proportion of students from low socio-economic backgrounds but a greater proportion of first- or second-generation immigrants.

Our results indicate that it is very important when it comes to interpreting findings about intergenerational mobility to critically assess how the data were collected. The minimum effort in survey studies should be to report openly about the level of non-response, as for example done by Björklund et al. (2006). If information is available about non-responders compared to responders, this can help to specify for which group the results hold. This is specifically important for policy effects, differentiated by sub-group.

The results also provide helpful insights into how to prevent biases already by designing the data collection. In the educational context, involving non-scientific institutions, such as schools, can increase acceptance in the targeted population. However, this also introduces an additional source of potential non-response bias. Our results show that institutional unit non-response poses an even greater threat to a study’s external validity than individual unit non-response.

## Appendix 3

Additional tables and graphs

See Tables 7, 8, 9, 10, 11, 12, 13, 14, 15, and 16 and Figs. 5, 6 and 7.

**Table 7:** Descriptive statistics for students without administrative data, baseline sample grade 6 (age 12)

Variable	Survey participation status									(1) vs. (2)	(1) vs. (3)	(2) vs .(3)
	(1) Survey participation			(2) School non-response			(3) Parental non-response					
	N	Mean	sd	N	Mean	sd	N	Mean	sd			
Age (in years in grade six)	510	12.26	.59	50	12.28	.64	227	12.38	.61		***	
Gender (fraction male)	536	.51	.50	73	.62	.49	247	.60	.49		******	
Low ses (fraction)	512	.19	.39	50	.02	.14	229	.21	.41	*******		*******
Migration background (fraction)	600	.19	.39	0	.	.	0	.	.	.	.	.
Highest parental education (1–5)	578	3.04	1.08	0	.	.	0	.	.	.	.	.
Total income (1–5)	0	.	.	0	.	.	0	.	.	.	.	.
Corrected HH income (1–5)	0	.	.	0	.	.	0	.	.	.	.	
Cito test score (500–550)	512	534.16	9.24	50	536.36	8.67	229	532.78	10.17		*****	******
Language test, grade 9 (stand.)	512	74.00	11.71	50	76.52	10.42	229	71.83	13.12		******	******
Mathematics test, grade 9 (stand.)	512	69.17	17.74	50	73.66	17.27	229	68.25	18.37	*****		*****
General cognitive ability (0–43)	575	31.70	4.99	**–**	**–**	**–**	195	31.41	4.74	.		.
	601			73			267					

The table reports the number of observations, the mean values and the standard deviations for the listed variables by response status on the parental survey in grade 6 at age 12 for all students for whom data could not be merged to administrative data from the income registry. The different sub-samples (1), (2), and (3) are mutually exclusive. Age is based on the students’ month-exact age at the beginning of grade 6, their final year in primary education. Gender takes on the value 1 for male students. “Low ses” is an indication used and provided by the school administration. Schools receive extra funding in case the proportion of students indicated as “low ses” exceeds a certain threshold. It is 1 if one or both of the parents did not complete secondary education (this indication is comparable to the “free lunch” indication in the US). A child is considered to have a migration background if at least one parent was not born in the Netherlands. Parental education level relies on a self-reported measure and is therefore only available if the parental survey was completed. It is coded in terms of the five levels defined in the International Standard Classification of Education (ISCED). Corrected income is reported on the household level in terms of quintiles relative to the entire Dutch population. It is based on the income register at 31 December 2008. The Cito test score reflects the overall score including sections on language, mathematics and study skills. The information provided on the language and mathematics sub-sections are reported in percent of questions answered correctly. Dots indicate that we do not have information on the variable for that particular group. A dash indicates that the variable was available for less than 10 students. In consequence, data protection guidelines of Statistics Netherlands prohibit the publication. Significance levels are reported as follows: .1*, .05**, and .01***

**Table 8:** Regression results for full sample with interactions, baseline sample 6 (age 12)

	Test scores					
	(1) Cito test	(2) Cito test	(3) Language (sub-section)	(4) Language (sub-section)	(5) Mathematics (sub-section)	(6) Mathematics (sub-section)
School (S) non-response	− .231* (.133)	− .231 (.145)	− .193 (.134)	− .193* (.110)	− .224* (.134)	− .224 (.143)
Parental (P) non-response	− .165* (.092)	− .165 (.106)	− .172* (.093)	− .172* (.099)	− .108 (.094)	− .108 (.096)
Income quintile	.147*** (.014)	.147*** (.014)	.143*** (.014)	.143*** (.014)	.124*** (.014)	.124*** (.014)
Income × S non-response	.086** (.038)	.086*** (.032)	.081** (.039)	.081*** (.029)	.073* (.039)	.073** (.033)
Income × P non-response	.007 (.029)	.007 (.031)	.013 (.029)	.013 (.030)	− .004 (.029)	− .004 (.028)
Controls	X	X	X	X	X	X
Clustered s. e.		X		X		X
R^2^	.152	.152	.136	.136	.130	.130
N	3699	3699	3699	3699	3699	3699

The table reports OLS regressions of children’s Cito test scores on parental income. The dependent variables are (1) overall Cito test scores and, respectively, sub-scores on the (2) language and (3) mathematics section, standardized to a mean of zero and a standard deviation of one. Income refers to corrected income. Each regression includes controls for a number of gender, students’ month-exact age, and indicators of low socio-economic status as well as of first- or second-generation immigrant status. Standard errors are reported in parentheses. Significance levels are reported as follows: .1*, .05**, and .01***

**Table 9:** Regression results for full sample with interactions, sample grade 9 (age 15)

	Test scores			
	(1) Language	(2) Language	(3) Mathematics	(4) Mathematics
Parental survey (P) non-response	.008 (.164)	.008 (.170)	− .480*** (.156)	− .480*** (.144)
Item (I) non-response	.389** (.179)	.389** (.186)	− .062 (.173)	− .062 (.164)
Income quintile	.134*** (.040)	.134*** (.043)	.074* (.038)	.074** (.036)
Income × P non-response	− .045 (.045)	− .045 (.047)	.057 (.043)	.057 (.041)
Income × I non-response	− .127** (.049)	− .127** (.051)	− .015 (.048)	− .015 (.046)
Controls	X	X	X	X
Clustered s.e.		X		X
R^2^	.096	.096	.104	.104
N	2120	2120	2094	2094

The table reports OLS regressions of children’s Cito test scores on parental income. The dependent variables are (1) overall Cito test scores and respectively sub-scores on the (2) language and (3) mathematics section, standardized to a mean of zero and a standard deviation of one. Income refers to corrected income. Each regression includes controls for a number of gender, students’ month-exact age and indicators of low socio-economic status as well as first- or second-generation immigrant status. Standard errors are reported in parentheses. Significance levels are reported as follows: .1*, .05**, and .01***

**Table 10:** Regression of school survey response on aggregate characteristics

	School survey response		
	(1)	(2)	(3)
Gender (fraction, males)	− .292 (1.194)	− .696 (1.321)	− .943 (1.381)
Low ses (fraction)	1.470 (1.481)	1.974 (1.829)	1.599 (1.834)
Migration background (fraction)	− .977 (1.595)	− .921 (1.765)	− .696 (1.864)
Income quintile 2 (fraction)	3.019 (2.114)	4.868* (1.555)	5.171* (2.676)
Income quintile 3 (fraction)	− .249 (1.788)	.191 (2.010)	.427 (2.162)
Income quintile 4 (fraction)	1.111 (1.835)	1.118 (2.088)	1.135 (2.214)
Income quintile 5 (fraction)	.548 (1.946)	.164 (2.166)	.348 (2.230)
Cito test score (500–550), average	− .007 (.061)	.006 (.065)	.004 (.066)
Cito test score (500–550), standard deviation	− .029 (.106)	− .036 (.117)	− .026 (.119)
Correlation: income quintile and Cito test score			− .502 (.673)
Location: Westelijke Mijnstreek		2.179** (.995)	2.353** (1.033)
Location: Parkstad		1.278 (.872)	1.412 (.891)
Location: Maastricht/Heuvelland		2.049*** (.933)	2.194** (.966)
Pseudo R^2^	.069	.163	.170
N	174	171	170

The table reports coefficients of Probit regressions. The dependent variable is school survey response. The independent variables include: the fraction of male students, the fraction of students with at least one parent who did not complete secondary education; the fraction of students who are first- or second-generation immigrants; the fraction of students, belonging to households of the indicated income quintile, based on corrected income; the average, the standard deviation of the Cito score and the correlation between income quintile and Cito test scores within each school; and indicators for school location (the baseline being north Limburg). Standard errors are reported in parentheses. Significance levels are reported as follows: .1*, .05**, and .01***

**Table 11:** Regression of parental survey response on individual characteristics

	Parental survey response					
	Baseline survey, grade 6 (age 12)			Sub-sample, grade 9 (age 15)		
	(1)	(2)	(3)	(4)	(5)	(6)
Gender (male = 1)	− .013 (.049)	− .013 (.049)	.002 (.055)	− .053 (.049)	− .008 (.049)	− .027 (.055)
Low ses	− .099 (.077)	− .072 (.077)	− .132 (.085)	− .431*** (.088)	− .377*** (.090)	− .440*** (.100)
Migration background	− .241*** (.069)	− .237*** (.069)	− .131* (.078)	− .327*** (.075)	− .355*** (.075)	− .329*** (.086)
Income quintile 2	.091 (.078)	.097 (.079)	.079 (.088)	.256*** (.086)	.262*** (.086)	.307*** (.094)
Income quintile 3	.071 (.081)	.062 (.081)	.045 (.090)	.159* (.085)	.151* (.086)	.085 (.095)
Income quintile 4	.103 (.082)	.087 (.083)	.041 (.092)	.354*** (.086)	.298*** (.087)	.306*** (.096)
Income quintile 5	.125 (.091)	.112 (.092)	.125 (.104)	.342*** (.092)	.272*** (.093)	.321*** (.103)
Cito test score (500−550)	.011*** (.003)	.012*** (.003)	.010*** (.003)	.014*** (.003)	− .012** (.005)	− .014** (.005)
Language and mathematics, grade 9					.247 (.163)	.267** (.181)
Track: pre-vocational (VMBO g/t)					.162* (.086)	.128 (.095)
Track: higher general (HAVO)					.533*** (.100)	.554*** (.110)
Track: academic (VWO)					.698*** (.121)	.739*** (.133)
Location: Westelijke Mijnstreek		− .493** (.232)	− .256 (.250)			− .093 (.172)
Location: Parkstad		− .507** (.230)	− .396 (.247)			− .096 (.170)
Location: Heuvelland		− .280 (.234)	− .174 (.252)			.005 (.173)
Location: Maastricht		− .502** (.238)	− .470* (.256)			− .180 (.184)
Speaking dialect well			.255*** (.059)			.131** (.058)
Pseudo R^2^	.015	.020	.031	.043	.055	.071
N	3328	3321	3034	2813	2813	2349

The table reports coefficients of Probit regressions. The dependent variable is parental survey response. The independent variables included for both samples are: a variable indicating male students, an indicator for students with at least one parent who did not complete secondary education; an indicator for students being first or second-generation immigrants; indicators for the quintile of corrected income the households belongs to; the student’s Cito test score; indicators for school location (with the baseline being north Limburg); and an indicator for speaking the local dialect well. Additionally, for the sample of secondary school students, a combined performance measure of the language and mathematics tests in grade 9, as well as indicators for the attended track (the baseline being the two lower pre-vocational tracks, VMBO b/k), are included. Standard errors are reported in parentheses. Significance levels are reported as follows: .1*, .05**, and .01***

**Table 12:** Regression of parental item response on individual characteristics

	Parental item response			
	(1)	(2)	(3)	(4)
Gender (male = 1)	− .135 (.104)	− .127 (.105)	− .061 (.116)	− .098 (.137)
Low ses	− .067 (.224)	− .034 (.230)	.041 (.257)	− .273 (.313)
Migration background	.053 (.175)	.044 (.175)	.017 (.195)	.061 (.233)
Income quintile 2	.130 (.201)	.113 (.202)	.200 (.222)	.333 (.275)
Income quintile 3	.310 (.199)	.299 (.199)	471** (.222)	.609** (.276)
Income quintile 4	.258 (.192)	.229 (.193)	.321 (.214)	.274 (.261)
Income quintile 5	.559*** (.200)	.534*** (.201)	.663*** (.223)	.972*** (.271)
Cito test score (500–550)	.018** (.007)	.010 (.011)	.012 (.012)	.010 (.014)
Language test, grade 9 (stand.)	− .040 (.061)	− .047 (.062)	− .040 (.068)	− .070 (.082)
Mathematics test, grade 9 (stand.)	− .020 (.061)	− .038 (.064)	− .045 (.071)	− .119 (.086)
Track: pre-vocational (VMBO g/t)		.145 (.208)	.169 (.223)	.085 (.276)
Track: higher general (HAVO)		.134 (.232)	.129 (.250)	.226 (.305)
Track: academic (VWO)		.299 (.274)	.202 (.297)	.272 (.363)
Location: Westelijke Mijnstreek			.122 (.326)	.489 (.403)
Location: Parkstad			.023 (.320)	.365 (.397)
Location: Heuvelland			− .078 (.325)	.046 (.399)
Location: Maastricht			− .054 (.353)	.107 (.430)
Speaking dialect well			− .061 (.123)	.096 (.148)
Response to first/online survey				1.982*** (.149)
Pseudo R^2^	.028	.030	.032	.350
N	650	650	548	548

The table reports coefficients of Probit regressions. The dependent variable is parental item response. The independent variables included are: a variable indicating male students, an indicator for students with at least one parent who did not complete secondary education; an indicator for students being first or second-generation immigrants; indicators for the quintile of corrected income the households belongs to; the student’s Cito test score; z-standardized performance measures of the language and mathematics tests in grade 9; indicators for the attended track (the baseline being the two lower pre-vocational tracks, VMBO b/k); indicators for school location (with the baseline being north Limburg); and an indicator for speaking the local dialect well. Standard errors are reported in parentheses. Significance levels are reported as follows: .1*, .05**, and .01***

**Table 13:** Relationship between students’ test scores and parental income, different samples (grade 6), no controls

	N	Coefficients for income quintile		
		(1) Cito test	(2) Language	(3) Mathematics
Full sample	3699	.213*** (.000)	.205*** (.000)	.171*** (.000)
Non-responding schools (NRS)	371	.284*** (.000)	.276*** (.000)	.228*** (.001)
Responding schools (RS)	3328	.204*** (.000)	.196*** (.000)	.165*** (.000)
Non-responding parents (NRP)	726	.217*** (.000)	.209*** (.000)	.168*** (.000)
Responding parents (RP)	2602	.196*** (.000)	.187*** (.000)	.160*** (.000)

Wald test on income coefficients	Hypothesis	p values		
School non-response	NRS = RS	.116	.035**	.173
Parental non-response	NRP = RP	.502	.474	.758

This table is similar to Table 5. Here, the estimations are performed without controls. Significance levels are reported as follows: .1*, .05**, and .01***

**Table 14:** Relationship between students’ test scores and parental income, different samples (grade 9), no controls

		Coefficients for income quintile			
		(1)		(2)	
		Language	N	Mathematics	N
Full sample		.122*** (.000)	2216	.152*** (.000)	2198
Survey non-responding parents (SNR)		.128*** (.000)	1247	.167*** (.000)	1241
Survey responding parents (SR)		.087*** (.001)	969	.093*** (.000)	957
Item non-responding parents (INR)		.042 (.191)	613	.080** (.011)	605
Survey, and item responding parents (IR)		164*** (.000)	356	.102*** (.005)	352

Wald test on income coefficients	Hypothesis	p values	
Parental survey non-response	SNR = SR	.225	.020**
Parental item non-response	INR = IR	.017**	.643

This table is similar to Table 6. Here, the estimations are performed without controls. Significance levels are reported as follows: .1*, .05**, and .01***

**Table 15:** Relationship between students’ test scores and parental income, different samples (grade 6), no migrants

	N	Coefficients for income quintile		
		(1)	(2)	(3)
		Cito test	Language	Mathematics
Full sample	3119	.154*** (.000)	.151*** (.000)	.132*** (.000)
Non-responding schools (NRS)	309	.198*** (.001)	.208*** (.000)	.182*** (.002)
Responding schools (RS)	2810	.148*** (.000)	.144*** (.000)	.126*** (.000)
Non-responding parents (NRP)	569	.151*** (.000)	.147*** (.000)	.115*** (.000)
Responding parents (RP)	2241	.145*** (.000)	.142*** (.000)	.127*** (.000)

Wald test on income coefficients	Hypothesis	p values		
School non-response	NRS = RS	.217	.112	.204
Parental non-response	NRP = RP	.860	.892	.742

This table is similar to Table 5. The estimations are performed on a sample excluding immigrants. Significance levels are reported as follows: .1*, .05**, and .01***

**Table 16:** Relationship between students’ test scores and parental income, different samples (grade 9), no migrants

	Coefficients for income quintile			
	(1)		(2)	
	Language	N	Mathematics	N
Full sample	.084*** (.000)	1817	.122*** (.000)	1779
Survey non-responding parents (SNR)	.091*** (.000)	976	.159*** (.000)	951
Survey responding parents (SR)	.069** (.014)	841	.071*** (.006)	828
Item non-responding parents (INR)	.027 (.422)	532	.065** (.052)	525
Survey, and item responding parents (IR)	.149*** (.001)	309	.081*** (.040)	303

Wald test on income coefficients	Hypothesis	p values	
Parental survey non-response	SNR = SR	.525	.010***
Parental item non-response	INR = IR	.027**	.748

This table is similar to Table 6. The estimations are performed on a sample excluding immigrants. Significance levels are reported as follows: .1*, .05**, and .01***

## Footnotes

1 Other studies use administrative data (see, e.g., Black et al. 2005). Black and Devereux (2011) provide an extensive review of this literature and the different empirical methods used.

2 Similar approaches to comparing survey data with full population register data have been taken in health science (Reijneveld and Stronks 1999; Søgaard et al. 2004) and by Micklewright and Schnepf (2006) to assess response bias in the PISA study in the UK.

3 Educational performance is an important predictor of later labor market outcomes (Murphy and Peltzman 2004). Education in general is considered an important mechanism to transmit power from one generation to the next (Wolff 2016).

4 Riphahn and Serfling (2005) explore this phenomenon and relate it to several demographic and interviewer characteristics. As Bielby et al. (1977) show, response errors may lead to overestimations of the racial gap and in turn to other severe misinterpretations.

5 Some studies have imputed missing responses based on geographically-aggregated data (e.g., Sabelhaus et al. 2015). Since Lillard et al. (1986) (for recent evidence see, e.g., Bee et al. 2016), questions regarding the validity of this approach have been raised.

6 For more evidence that survey response is positively related to income see, e.g., Martikainen et al. (2007), Porter and Whitcomb (2005), or Turrell et al. (2003).

7 Although misreporting of income is not the main focus of our analysis, our results do relate to this issue as well. Research on measurement error regarding earnings has linked survey data to administrative record data (see, e.g., the survey by Bound et al. 2001). One of our analyses compares the correlation between the administrative measure of income and educational outcomes with the correlation between a self-reported measure of income and educational outcomes. The results show that the latter is attenuated relative to the former. This is in line with the analysis by Hariri and Lassen (2017) who show that high-income earners tend to overstate their income. Similar to our analysis, they also compare administrative measures of income with self-reported measures. The context of their study differs from ours, however, in the sense that they study the relationship between income and political attitude.

8 Additional information about the monitor can be found in the appendix.

9 This compares very well to the response rates or survival rates in the articles by Chevalier et al. (2013) and by Plug and Vijverberg (2005), as can be seen from technical reports on the underlying surveys (EUROSTAT 2009; Hauser 2005). The response rate of 58% reported by Björklund et al. (2006) is somewhat lower. The only higher response rate in any of the articles on the estimated relationship between parental income and child schooling cited above amounts to 87% in the National Longitudinal Survey of Youth (NLSY) used by Blau (1999).

10 In this wave of the data, we observe that at least one parent from each school completed the survey. This supports our claim of institutional unit non-response based on observing a zero response rate at the school level. The overall low response rate can be explained by the fact that the initial invitation letter only included a link to an online survey. Only the reminder letter included a paper version. 52.8% of participants completed the paper survey.

11 As a comparison, Bee et al. (2016) manage to link between 76 and 79% of households to tax administration data in the US context. They identify non-filing of tax returns as a major reason for this incomplete link.

12 Officially, the test is called “Cito Eindtoets.” It comprises three parts: language, mathematics, and study skills. It is designed and also graded externally by the central institute for test development (Centraal Instituut voor Toetsontwikkeling, or Cito).

13 Tracks are important in the Dutch schooling system. After primary school, around age 13, children are assigned to different secondary school tracks. These tracks lead to specific qualifications for the labor market. Even if the teacher deviates from the recommendation suggested by the final assessment test result, some secondary schools only accept students with a score above a certain threshold (Borghans et al. 2015). During the first three years after the transition to secondary school, a quarter of the children changes track at least once. Of these, 40% change to a higher track (Inspectie van het Onderwijs 2014).

14 The tests comprise questions used in the tests of the Programme for International Student Assessment (PISA) as well as the Dutch cohort study on educational careers for students aged 5–18 (Cohort Onderzoek OnderwijsLoopbanen, COOL^5–18^).

15 The two upper tracks are VWO, which qualifies students for university, and HAVO, which provides access to universities of applied sciences. The other two tracks represent the two levels within the pre-vocational education (VMBO) system.

16 In a robustness check, we compare the results based on the raw income data. These are qualitatively similar.

17 The schools are required to administer a test to primary school leavers. With about 86% coverage in 2013, the Cito test we use in the analysis is the most common one (Inspectie van het Onderwijs 2014). Therefore, this is the best comparative measure available in this context.

18 These differences are significant. Results are available on request.

19 Earlier studies on the relationship between parental income and child schooling also use controls for basic demographic characteristics of the child (Chevalier et al. 2013) and in some cases additional child, parent, and household characteristics (Blau 1999; Plug and Vijverberg 2005).

20 As indicated earlier, the latter variable is mainly based on whether the parents have a particularly low educational background. It is our best available proxy for parental education background for the full sample, including non-responding parents.

21 The comparison between the coefficients of the NRS and RS samples provides a measure of whether and how institutional unit non-response biases the estimate (see appendix for a discussion of the estimates).

22 All control variables, gender, age, and “low ses” (Socio-Economic Status) are significant. The coefficient for low socio-economic background has a negative sign. Higher age is associated with a lower Cito score, most likely because we are not able to control for grade retention. In addition, male students perform slightly better.

23 The difficulty level of the tests differs across schooling tracks, but with an overlap in questions. To place students on one scale across schooling tracks we run a two-parameter item response theory model.

24 We further discuss this in Sect. 5.2. An alternative approach, using interaction terms in a regression (see Table 9), confirms the overestimation driven by item non-response. Applied to the difference between the lowest and the highest income quintile, the induced bias amounts to more than half a standard deviation in the language achievement test. It almost completely offsets the estimated positive association between parental income and performance regarding the language test.

25 Examples of individuals with low income but otherwise favorable characteristics would be a university graduate with a degree in philosophy who works as a taxi driver, or individuals who are still completing their education, such as PhD students.

26 The coefficient indicating households in the middle income quintile most likely only becomes significant due to the sample restriction from column (2) to column (3). Restricting the regressions in columns (1) and (2) in the same way leads to significant coefficients as well.
